# Flexibility is everything: prey capture throughout the seasonal habitat switches in the smooth newt *Lissotriton vulgaris*

**DOI:** 10.1007/s13127-014-0187-1

**Published:** 2014-10-31

**Authors:** Egon Heiss, Peter Aerts, Sam Van Wassenbergh

**Affiliations:** 1 Institute of Systematic Zoology and Evolutionary Biology, Friedrich-Schiller-University Jena, Erbertstr. 1, 07743 Jena, Germany; 2 Department of Biology, University of Antwerp, Universiteitsplein 1, 2610 Antwerp, Belgium; 3 Department of Integrative Zoology, University of Vienna, Althanstr. 14, 1090 Vienna, Austria; 4 Department of Movement and Sports Sciences, Ghent University, Watersportlaan 2, 9000 Ghent, Belgium; 5 Evolutionary Morphology of Vertebrates, Ghent University, K.L. Ledeganckstraat 35, 9000 Ghent, Belgium

**Keywords:** Amphibia, Feeding biology, Kinematics, Behavioral plasticity, Salamanader

## Abstract

Transitions between aquatic and terrestrial habitats are significant steps in vertebrate evolution. Due to the different biophysical demands on the whole organism in water and air, such transitions require major changes of many physiological functions, including feeding. Accordingly, the capability to modulate the pre-programmed chain of prey-capture movements might be essential to maintain performance in a new environment. Newts are of special interest in this regard as they show a multiphasic lifestyle where adults change seasonally between an aquatic and a terrestrial stage. For instance, the Alpine newt is capable of using tongue prehension to feed on land only when in the terrestrial stage, but still manages to suction feed if immersed whilst in terrestrial stage. During the aquatic stage, terrestrial feeding always involved grasping prey by the jaws. Here, we show that this seasonal shift in feeding behavior is also present in a species with a shorter terrestrial stage, the smooth newt *Lissotriton vulgaris*. Behavioral variability increases when animals change from aquatic to terrestrial strikes in the aquatic stage, but prey-capture movements seem to be generally well-coordinated across the feeding modes. Only suction feeding in the terrestrial stage was seldom performed and appeared uncoordinated. Our results indicate that newts exhibit a high degree of seasonal flexibility of the prey-capture behavior. The similarity between movement patterns of suction feeding and terrestrial feeding suggests that only relatively subtle neuromotoric adjustments to the ancestral, suction-feeding motor program are required to successfully feed in the new environment.

## Introduction

Transitions from an aquatic to a terrestrial lifestyle require changes in form and function of almost every organ system (Denny 1993; Vogel 1994; Carroll 2009; Stayton 2011; Clack 2012). Amongst others, changes are required in terms of osmoregulation, sensory system, locomotion, respiration, as well as for feeding (e.g., Ashley-Ross et al. 2013). The ability to capture and transport food both in aquatic and terrestrial environments is most challenging for the organism as the mechanical demands on the whole feeding biology are different (Deban 2003). Although animals living on the edge of both realms would greatly benefit from exploiting food sources from both environments, only few vertebrates are capable of coping with the mechanical problems of performing efficiently in both water and air. Most aquatic predators use a prey-capture mechanism referred to as suction feeding where a fast oropharyngeal volume expansion generates a suction flow that drives prey and surrounding water to flow into the gaping mouth (Alexander 1967; Muller and Osse 1984; van Leeuwen and Muller 1984; Lauder 1985). The engulfed water is then released through the gill openings (unidirectional flow) in virtually all fish species and larval amphibians, or through the slightly opened mouth (bidirectional flow) in forms with closed gill slits (Lauder and Shaffer 1986). Due to the low viscosity and density of air, a prey-capture mode relying on suction does not work on land (Bramble and Wake 1985; Herrel et al. 2012). In general, terrestrial prey-capture strategies rely on grasping by the jaws (i.e., jaw prehension) or by the tongue (i.e., tongue prehension; Bramble and Wake 1985; Larsen et al. 1996; Schwenk 2000).

Because of this clear difference in prey-capture strategy between specialized aquatic and terrestrial vertebrates, it is assumed that amphibious forms that regularly feed in both environments perform sub-optimally in at least one environment. These amphibious vertebrates include some species of fishes (Sponder and Lauder 1981; Van Wassenbergh et al. 2006; Van Wassenbergh 2013), aquatic salamanders (Lauder and Shaffer 1988; Miller and Larsen 1990) and aquatic turtles (Summers et al. 1998; Natchev et al. 2009, 2010; Stayton 2011) that occasionally feed on land using a slightly modified aquatic prey-strike pattern. However, most terrestrial vertebrates specialized to the terrestrial conditions have lost the ability to capture prey in water (Schwenk 2000). The best solution for amphibious vertebrates would be to change their behavior and use two different media-dependent strategies (Heiss et al. 2013a). Accordingly, it seems that the ability to modulate behavior, i.e., the ability to change the neuromotor program of an established chain of movements to gain a new behavior, is one of the key prerequisites for species invading new environments.

Newts are of special interest in studying the role of behavioral flexibility in environmental transitions as they exhibit a unique multiphasic lifestyle where they change seasonally between aquatic and terrestrial habitats. These seasonal shifts between habitats induce notable changes of the whole organism and result in an aquatic and a terrestrial stage with two distinct morphotypes (Matthes 1934; Halliday 1974; Nöllert and Nöllert 1992; Griffiths 1997; Warburg and Rosenberg 1997; Denoël 2004). The most obvious differences between the two morphotypes concern presence (aquatic morphotype) or absence (terrestrial morphotype) of tail fins and labial lobes, as well as changes in thickness and texture of the skin (Matthes 1934; Nöllert and Nöllert 1992; Duellman and Trueb 1994; Thiesmeier and Schulte 2010). However, except for the outgrowth and reduction of labial lobes (Matthes 1934), no morphological changes of the feeding system have been described during seasonally induced environmental transitions in newts.

Recently, it was hypothesized that habitat shifts require changes in prey capture behavior of the Alpine newts (*Ichthyosaura alpestris*) in order to keep acceptable prey capture performance in the respective medium (Heiss et al. 2013a). Unexpectedly, that study showed that the changes in habitat only affect the terrestrial feeding pattern and that aquatic feeding does not change, regardless the current seasonal stage. When in the aquatic phase, animals feeding on land used a markedly similar behavior as used in water. In contrast, when in the terrestrial phase, a terrestrial strike showed distinct differences from a strike performed underwater and was characterized by a quick protrusion and subsequent retraction of the tongue. However, analyses of the terrestrial feeding kinematics indicated that terrestrial feeding movement patterns can be largely derived from aquatic feeding patterns. Consequently, terrestrial feeding in newts may have evolved from relatively small modulations and recombination of the ancestral aquatic mode (Heiss et al. 2013a).

While these findings might explain how a novel prey-capture behavior can evolve in the course of an environmental transition, it has only been shown for a single species (*I. alpestris*, see Heiss et al. 2013a). The behavioral plasticity could, therefore, be a trait unique to the Alpine newt. A broader approach is necessary to show whether modulation and recombination of feeding movement patterns is indeed a common feature in newts with a multiphasic lifestyle. To do so, we here perform a kinematic analysis on the smooth newt (*Lissotriton vulgaris*), a member of the sister-clade (*Lissotriton*) of the Alpine newt (Weisrock et al. 2006; Zhang et al. 2008). Both newt species show a multiphasic lifestyle and often live sympatrically (Nöllert and Nöllert 1992). However, the aquatic stage of the smooth newt is generally shorter compared to the Alpine newt (Griffiths 1997) and we predict that the reduction in aquatic stage duration will reduce the behavioral plasticity in its prey-capture performance across the two stages. Specifically, we hypothesize that the shorter aquatic stage in the smooth newt and its predominant terrestrial lifestyle might negatively influence the aquatic capture performance, especially during its terrestrial stage.

A second objective of the current study is to show how the seasonal environmental transitions by *L. vulgaris* affect its behavioral stereotypy and coordination of movements. Specifically, we hypothesize that prey-capture kinematics of *L. vulgaris* will be more stereotyped when feeding in the prevalent feeding modes of aquatic feeding in the aquatic stage and terrestrial feeding in the terrestrial stage compared to the reciprocal modes terrestrial feeding in the aquatic stage and aquatic feeding in the terrestrial stage. However, when capturing prey in the reciprocal modes (striking on land when in the aquatic stage and vice versa) newts are probably faced with the need to adjust and continue to fine-tune their movements for strikes to be successful. As a result, they might show higher degrees of variability. As a coordinated interplay of jaws and hyobranchial movements is advantageous for a successful strike at the prey (Ferry-Graham and Lauder 2001; Wainwright et al. 2008) and probably needs time to be fine-tuned after the transition, we predict the coordination of hyobranchial and jaw movements to be higher when feeding in the medium of the corresponding stage (e.g., aquatic strike in the aquatic stage) compared to the reciprocal modes (e.g., terrestrial strike in the aquatic stage). By studying the prey-capture behavior of *L. vulgaris* in the four possible modes, comparing these data with recently published data on the Alpine newt (Heiss et al. 2013a), and quantifying the influence of environmental transitions on both behavioral variation and movement coordination, we aim for a better understanding of how environmental transitions are achieved in newts and vertebrates in general.

## Material and methods

### Study animals

Twenty individuals of *L. vulgaris* were collected during their aquatic stage between April and June 2011 in Lower Austria, Austria with collection permission RU5-BE-18/022-2011 granted by the local government of Lower Austria. All specimens were transferred to the Laboratory of Functional Morphology, University of Antwerp (Belgium), where experiments were performed. The animals were kept in large tanks with water levels of 15 cm and an easily accessible land part. The animals were fed twice a week with a variety of red mosquito larvae (chironomids), firebrats (*Thermobia domestica*), and maggots (*Lucilia* sp.). Animal keeping and experiments were approved by the Ethical Commission for Animal Experiments of the University of Antwerp (code: 2010-36).

### High-speed video recording

To record feeding kinematics, animals were trained to feed in a small glass aquarium (ground area, 12 × 30 cm; height, 20 cm) where they were filmed with two digital high-speed cameras (Redlake MotionScopeM3 and Redlake Motion-Pro HR1000a; Redlake Digital Imaging Systems, IDT Vision, Tallahassee, FL, USA) in lateral and ventral views at a frame rate of 500 Hz. Animals were deprived of food for 4 days before experiments.

Four infrared spotlights were used as light sources for videography. To avoid distortive effects of different prey types on the prey capture behavior (Maglia and Pyles 1995; Deban 1997), we used living maggots as standardized prey items. Maggots were also used because they are a natural prey and all newts showed strong reactions towards them and readily fed on them.

The experiments were performed when the newts were in the aquatic stage and again after they had changed to the terrestrial stage. Animals were given 4 weeks of time after the change of stages to accommodate to the new habitat.

Two environmental situations in two stages were recorded as follows: (a) aquatic feeding in the aquatic stage, (b) terrestrial feeding in the aquatic stage, (c) terrestrial feeding in the terrestrial stage, and (d) aquatic feeding in the terrestrial stage.

For filming aquatic feeding in the aquatic stage, prey was offered in front of the animals in the experimental aquarium with 5 cm water level. The same setup was used to record aquatic feeding in the terrestrial stage. For filming terrestrial feeding in the aquatic stage, a Plexiglas ramp was placed into the aquarium with 5 cm water level and the newts were then slowly lured out of the water over the ramp to capture the prey offered on land. For filming terrestrial feeding in the terrestrial stage, prey was offered in front of the animals in the same aquarium but without water. The ventral view recordings were performed to determine lateral expansion movements of the head during prey capture. However, as no significant lateral movements could be measured, they were excluded from further analyses.

### Kinematics

For kinematic analyses, we selected the lateral recordings of feeding trials from ten similarly sized individuals (total length of 85.8 ± 5.0 mm) that together provided strike recordings in the four following modes: aquatic feeding in the aquatic stage, terrestrial feeding in the aquatic stage, aquatic feeding in the terrestrial stage, and terrestrial feeding in the terrestrial stage (see Table 1). For each individual feeding in the specific mode, five repetitions were used, resulting in a total of 80 recordings for further analysis.

**Table 1:** Overview of individual newts that together provided high-speed recordings in the four feeding modes

	Aquatic stage		Terrestrial stage	
	Aquatic feeding	Terrestrial feeding	Terrestrial feeding	Aquatic feeding
Individual	A, B, C, D, E	A, B, C, D, E	A, F, G, H, I	K

**Fig. 1:**
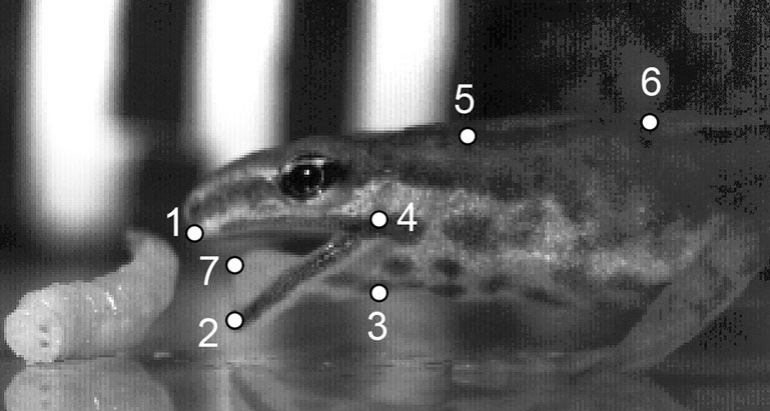
Landmarks used for the kinematic analyses. *1* upper jaw tip, *2* lower jaw tip, *3* hyoid (throat), *4* jaw joint, *5* nape, *6* dorsal trunk reference, *7* tongue tip (only digitized when visible)

The horizontal (*x* axis) and vertical (*y* axis) coordinates of defined landmarks (Fig. 1) were tracked frame by frame using SIMI-MatchiX software (SIMI Reality Motion Systems, Germany). Our landmarks were based on those used by other studies on salamander prey capture (e.g., Shaffer and Lauder 1985; Reilly 1995, 1996; Deban 1997; Deban and Marks 2002; Deban and O’Reilly 2005; Heiss et al. 2013a, b) to allow direct comparisons of kinematics. According to the 2D displacements of the landmarks, we calculated the following movements: jaw movement (distance between the tips of the upper and the lower jaw), head rotation (dorsoventral angle displacements of head relative to the trunk), hyoid depression (distance between jaw joint and throat where maximum depression occurs), and tongue movements (distance between tongue-tip and jaw joint) only in the terrestrial feeding mode of the terrestrial morphotype. From these kinematic profiles, 12 kinematic variables that summarize the kinematics of a whole prey-capture event were determined in analogy with previous research on prey-capture biomechanics in salamanders (e.g., Shaffer and Lauder 1985; Reilly 1995, 1996; Deban 1997; Deban and Marks 2002; Deban and O’Reilly 2005; Heiss et al. 2013a, b). These variables were as follows: (1) duration of gape opening (i.e., time from start of mouth opening till maximum gape), (2) duration of gape closing (i.e., time from maximum gape till mouth closing), (3) maximum gape (i.e., maximum distance between upper and lower jaw tips), (4) velocity of gape opening (i.e., mean velocity of mouth opening calculated as derivative of gape change), (5) velocity of gape closing (i.e., mean velocity of mouth closing calculated as derivative of gape change), (7) duration of hyoid depression (i.e., time from start of hyoid depression till maximum hyoid deflection), (8) maximum hyoid depression (i.e., maximum distance between jaw joint and ventral hyoid deflection), (9) velocity of hyoid depression (i.e., mean velocity of ventral hyoid deflection calculated as a derivative of hyoid deflection), (10) maximum head elevation (i.e., maximum angle of head relative to trunk), (11) duration of head elevation (i.e., time from the start of head elevation till maximum head deflection), and (12) velocity of head elevation (i.e., mean angular velocity of head elevation calculated as a derivative of head rotation).

### Statistics

#### Flexibility (*sensu* Wainwright et al. 2008)

After calculating descriptive statistics for each variable and individual, we checked for normal distribution of their residuals by using the Kolmogorov–Smirnov test; if they were not normally distributed, we log10 transformed those variables, after which their residuals were normally distributed. Then, in order to test the flexibility of behavior, we performed a multivariate ANOVA (MANOVA) where “feeding mode” was treated as fixed factor. Additional MANOVAs were performed to test for differences between individuals in all three feeding modes where data from five individuals were available (respectively, with “individual” as fixed factor). The four MANOVASs had to be calculated separately in order to account for not meeting full-factorial conditions with our data (not the same individuals were present in all four modes, see Table 1). In order to account for running multiple tests, the simultaneous Bonferroni correction was used to adjust significance levels to *P* ≤ 0.0042 for all resulting ANOVAs. Additionally, we performed a principal component analysis to show the effects of both the feeding mode and the individuals on the total variance of the feeding behavior.

**Table 2:** Descriptive statistics of kinematic variables in the four different feeding modes and subsequent ANOVAs testing differences between the four feeding modes and differences between individuals in the three feeding modes where several individuals could be tested

	Aquatic stage		Terrestrial stage		ANOVAs			
Variable	Suction feeding	Jaw prehension	Suction feeding	Tongue prehension	Mode	Individual		
						Suction feeding (aquatic stage)	Jaw prehension (aquatic stage)	Tongue prehension (terrestrial stage)
	Mean ± SD	Mean ± SD	Mean ± SD	Mean ± SD	*F* ratio	*F* ratio	*F* ratio	*F* ratio
Duration of gape opening (ms)	30 ± 6	65 ± 21	32 ± 6	113 ± 31	125.6*	0.5	2.8	5.6*
Duration of gape closing (ms)	21 ± 4	40 ± 11	28 ± 3	34 ± 9	22.8*	1.8	0.9	0.9
Maximum gape (mm)	4.5 ± 0.8	6.7 ± 0.7	4.4 ± 0.7	5.2 ± 0.8	38.1*	1.3	4.2	1.9
Velocity of gape opening (ms^−1^)	0.15 ± 0.03	0.11 ± 0.03	0.14 ± 0.03	0.05 ± 0.01	74.5*	1	5	2.4
Velocity of gape closing (ms^−1^)	0.2 ± 0.1	0.16 ± 0.05	0.15 ± 0.02	0.13 ± 0.05	7*	5.6*	1.7	2.8
Time to start hyoid depression (ms)	9.4 ± 5	13.5 ± 13.8	16 ± 6	76 ± 15	68.8*	0.4	3.1	6.1*
Duration of hyoid depression (ms)	25 ± 6	59 ± 16	25 ± 3	63 ± 25	29.1*	6.6*	1.1	1.2
Maximum hyoid depression (mm)	4 ± 0.9	3.6 ± 0.9	2.8 ± 0.5	1.6 ± 0.5	47.4*	1.7	3.9	0.3
Velocity of hyoid depression (ms^−1^)	0.17 ± 0.05	0.06 ± 0.02	0.12 ± 0.03	0.03 ± 0.01	82.2*	10.1*	2.1	1.2
Maximum head elevation (°)	15 ± 5	13 ± 6	7.6 ± 4.2	20.3 ± 5.4	12.2*	6.9*	1.9	10*
Duration of head elevation (ms)	28 ± 8	45 ± 24	26 ± 13	113 ± 28	74.6*	1.3	2.6	5.2
Velocity of head elevation (°s^−1^)	542 ± 185	313 ± 167	292 ± 126	181 ± 37	26.8*	2.8	2.1	4.1

Significance level was adjusted to *P* ≤ 0.0042 after simultaneous Bonferroni correction, and the asterisk (*) indicates *F* ratios with significant *P* value (i.e., *P* ≤ 0.0042)

Wainwright et al. (2008) defined “flexibility” as “the extent to which the behavior is altered in response to a change in stimulus” or “the ability of an organism to alter its behavior across experimental treatments.” We mainly follow this definition, but given the multiphasic lifestyle in newts which is coupled to some functional-morphological changes during the environmental transitions, it is important to mention that “flexibility” in our study is no longer pure “behavioral flexibility,” but also includes to some degree functional-morphological plasticity.

#### Behavioral variation (*sensu* Wainwright et al. 2008)

To test for behavioral variation within the four feeding modes, we calculated the coefficient of variation (CV), as standard deviation divided by the mean, for each of the 12 variables for each individual in the four feeding modes. Next, we checked for normal distribution and as values where normally distributed, performed a fractional factorial designed analysis of variance (ANOVA) with mode and individual treated as fixed factors. A Bonferroni post hoc test was performed to test for differences of the CV between feeding modes.

#### Coordination of movements (*sensu* Wainwright et al. 2008)

The coordination between movements was calculated as bivariate correlations between kinematic variables. We checked for correlations between gape and hyoid movement variables because gape and hyoid movements are mechanically uncoupled from each other (e.g., Deban and Wake 2000; Wake and Deban 2000) and the correlation of their variables would indicate active coordination (Wainwright et al. 2008). Accordingly, if movement variables were significantly correlated (Pearson correlation) both chronologically (time) and in magnitude (distance), they were considered as “well-coordinated movements.” When movement variables only correlated chronologically or in magnitude, they were considered as “less-coordinated,” and when movements did not correlate at all, as “not-coordinated.” All statistical analyses were performed using Microsoft Excel 2010 (Microsoft, USA) and SPSS Statistics 20 software package (IBM, USA).

## Results

### Kinematics of aquatic prey capture

**Fig. 2:**
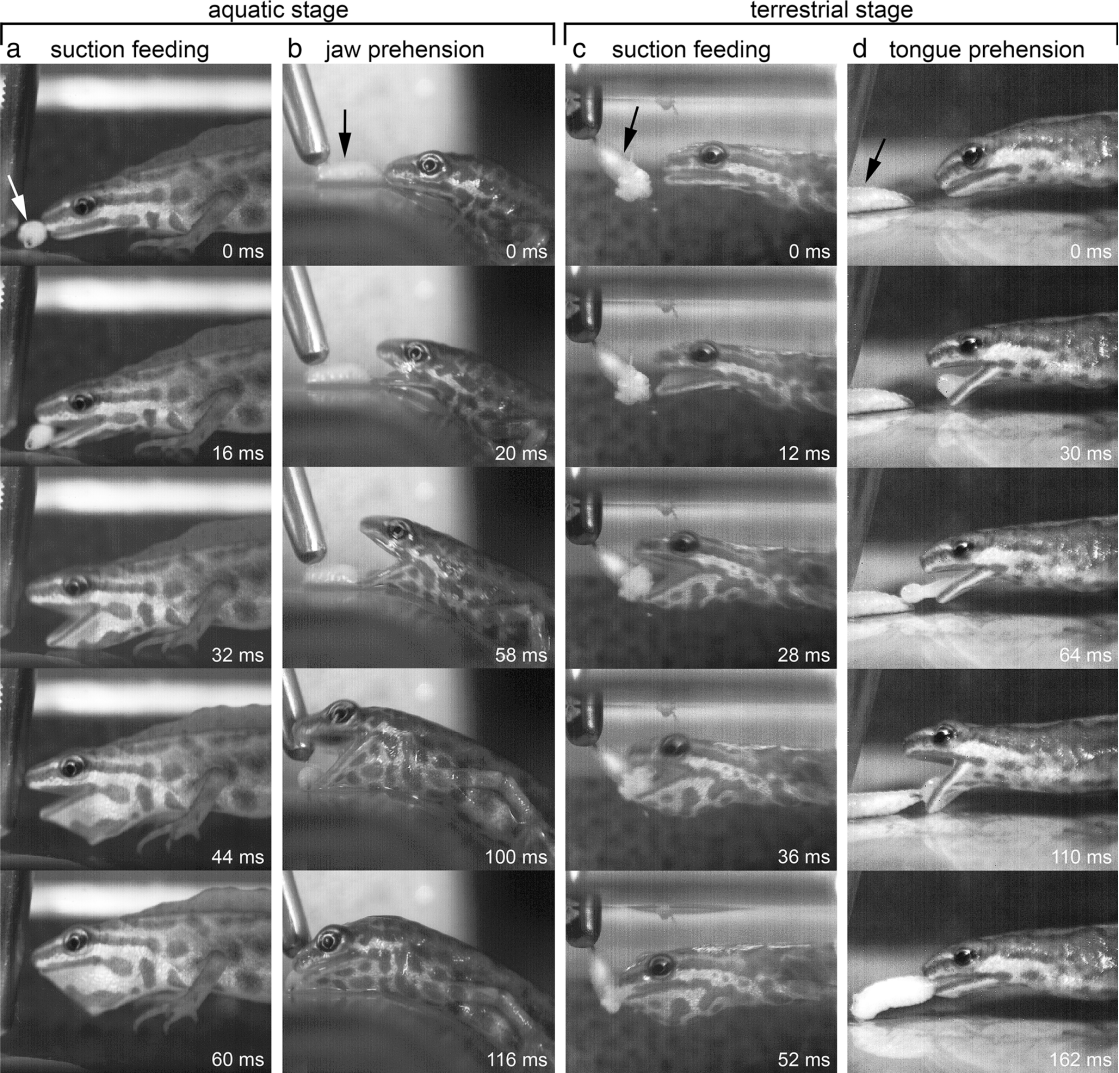
Frame shots showing the four feeding modes in the smooth newt. In the aquatic stage: **a** suction feeding under water and **b** jaw prehension on land. In the terrestrial stage: **c** suction feeding under water and **d** tongue prehension on land. The prey (maggot) is indicated by the *arrow*. Note the similar prey capture movements in the first three modes: suction feeding in the aquatic stage, jaw prehension, and suction feeding in the terrestrial stage, and the distinct pattern in the last mode: tongue prehension, where prey is captured by the tongue

**Fig. 3:**
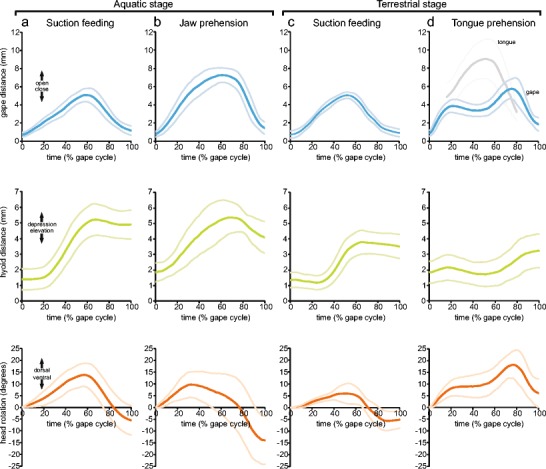
Kinematic profiles of the four feeding modes. Kinematic means (*dark* and *bold curves*) ± SD (*pale* and *slim curves*) of gape (*blu*e), hyoid (*green*), head rotation (*orange*), and tongue movement (*gray*, only shown in **d**). The time scale on the *x* axes is normalized as percentages of total gape cycle, but see Table 2 for absolute values

When capturing prey underwater in the aquatic stage, *L. vulgaris* approached prey and then sucked it up by an anterior to posterior oropharyngeal expansion wave (Figs. 2a and 3a). The strike at the prey started with mouth opening, achieved by dorsal head rotation and lower jaw depression. The gape reached its peak of 4.5 ± 0.8 mm (mean ± SD) 30 ± 6 ms after the onset of mouth opening, immediately after which the mouth started closing. Hyoid depression started with a short delay of 9.4 ± 5.0 ms after the onset of mouth opening and reached its ventral depression peak of 4.0 ± 0.9 mm after 25 ± 6 ms, almost simultaneously with the peak gape (see section on correlations below). Prey was sucked in before jaw closing started (Figs. 2a and 3a). The angle of the skull relative to the longitudinal body axis was smaller after jaws were closed (end of gape cycle) than before jaw opening started (start of gape cycle). The whole gape cycle was described by a bell-shaped curve and lasted 51.4 ± 7.5 ms (Table 2; Figs. 2a and 3a).

The movements of the one animal that captured prey underwater in the terrestrial stage were slightly slower compared to those in the aquatic stage. Otherwise, the kinematic profile of aquatic feeding in the terrestrial stage was very similar to the aquatic feeding profile in the aquatic stage as described above (see Table 2; Figs. 2c and 3c and statistics below) and both feeding modes can be referred to as suction feeding.

### Kinematics of terrestrial prey capture

The terrestrial prey capture in the terrestrial stage again started with a slow approach to the prey. Then, the mouth was opened and the gape reached an initial peak of 4.0 ± 0.7 mm after 30.5 ± 6.8 ms (Figs. 2d and 3d). During this first jaw-opening phase, the tongue was slowly protracted to the margins of the lower jaw. Next, the mouth was slightly closed again and reached a local minimum (Figs. 2d and 3d). At the same time, the tongue was projected out of the mouth, reaching its maximum protraction of 11.6 ± 2.1 mm (tongue tip relative to jaw joint) and contacting the prey. Then, when the tongue with adhering prey started retracting, the gape increased again and reached its second peak of 5.2 ± 0.8 mm after 113 ± 31 ms, prey was brought behind the margins of the jaws and engulfed (Figs. 2d and 3d) and jaws started closing. The two-peaked kinematic gape profile, with the first peak being always lower than the second peak, lasted 146 ± 36 ms (Table 2; Fig. 3d).

At the onset of the prey-capture when the mouth started opening, the hyoid was slightly depressed and then elevated after the gape reached its first peak (Fig. 3d). The peak of the hyoid elevation coincided with the local gape minimum which appeared between both gape peaks, and then the hyoid was depressed again till mouth was closed (Fig. 3d and section on coordination of movements below). In sum, the tongue-based prey capture mode in the terrestrial stage can be referred to as “tongue prehension.”

For capturing prey on land in the aquatic stage, newts had to emerge onto land through a shallow ramp where prey was offered. The strike at the prey started with jaw opening followed by hyoid depression (Figs. 2b and 3b). The gape reached its peak of 6.7 ± 0.9 mm after 65 ± 21 ms, resulting in a gape profile with a bell-shaped curve. Hyoid depression started after the onset of mouth opening and reached its maximum ventral deflection of 3.6 ± 0.9 mm after 59 ± 16 ms, after which the hyoid was elevated again (Table 2; Fig. 3b). Prey was seized by the closing jaws and was brought back to the water for further intraoral transport and swallowing. The angle of the skull relative to the longitudinal body axis was smaller after the jaws were closed than before jaw opening started and the whole gape cycle lasted 106 ± 26 ms (Table 2; Fig. 3b). Tongue protractions were not observed to be involved in this prey-capture mode as prey was always grasped by the jaws. Accordingly, terrestrial prey-capture in the aquatic stage can be referred to as “jaw prehension.”

### Flexibility of behavior: kinematic differences between the four feeding modes

The MANOVA with mode treated as fixed factor showed a highly significant overall difference between the four feeding-modes (Wilks’ lambda *F* = 16.26, *P* < 0.001). The subsequent series of ANOVAs showed that all 12 variables tested were in fact significantly different between modes (Table 2). The posthoc test (Tukey-HSD) further detailed the differences between the single feeding-modes. Suction feeding in the aquatic stage differed significantly from terrestrial feeding in the terrestrial stage (i.e., tongue prehension) in all 12 variables and from terrestrial feeding in the aquatic stage (i.e., jaw prension) in eight out of the 12 variables. By contrast, suction feeding in both stages differed in only two variables. The tongue prehension mode not only differed clearly from suction feeding in the aquatic stage (see above), but also from suction feeding in the terrestrial stage (concerning eight variables) and from jaw prehension (concerning ten variables). Both reciprocal modes, i.e., jaw prehension and suction feeding in the terrestrial stage differed in six out of the 12 variables tested.

In order to account for individual variation, we separately tested for differences between individuals in the three feeding modes where data were available for five individuals each (i.e., suction feeding in the terrestrial stage had to be excluded because only one individual fed in that mode).

The differences between individuals were significant in the modes suction feeding in the aquatic stage (Wilks’ lambda *F* = 1.9; *P* = 0.02) and in tongue prehension (Wilks’ lambda *F* = 3.9; *P* < 0.001), but showed no significant difference in jaw prehension (Wilks’ lambda *F* = 1.4; *P* = 0.13). The series of ANOVAs further showed that the statistically significant difference between individuals in suction feeding in the aquatic stage was due to significant differences between four out of the 12 variables (Table 2) and differences between individuals in tongue prehension due to three significantly different variables (Table 2).

**Fig. 4:**
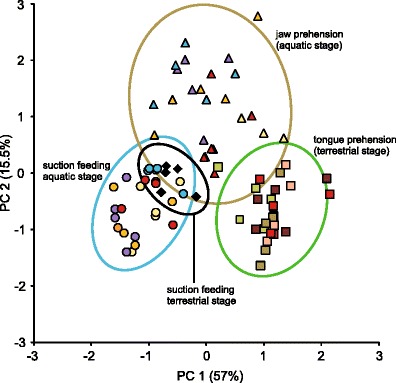
Scatter plot of the first two principal components. Principal component 1 (*PC1*) and principal component 2 (*PC2*) are derived from the 12 kinematic variables to illustrate the relationship among kinematic patterns for the four feeding modes coded by symbols and the ten individuals coded by color. Each *data point* represents one feeding event, and the *ellipses* indicate 95 % confidence interval in the four feeding modes. PC1 explains 57 % and PC2 explains 15.5 % of the total variance. See Table 3 for complete loadings of each principal component

Figure 4 shows the multivariate dispersion of kinematics among the four feeding modes and the ten individuals on the first two principal components, and the loadings of the variables on components 1–3 are given in Table 3. Suction feeding in the aquatic stage almost entirely overlaps in kinematic space with suction feeding in the terrestrial stage. Jaw prehension shows a distinct distribution pattern but overlaps with all other three behaviors to a certain degree. In fact, jaw prehension lies between suction feeding in both stages and tongue prehension. Tongue prehension shows no overlapping area with suction feeding in both stages but a small area of kinematic space is shared with jaw prehension. By contrast, the dispersion of individuals in kinematic space broadly overlaps and is clearly related to the feeding modes (Fig. 4: especially shown by the individual coded in red).

**Table 3:** Loadings of the 12 variables to the first three principal components

Variable	PC1	PC2	PC3
Duration of gape opening	0.956	0.058	0.208
Duration of gape closing	0.589	0.62	−0.006
Maximum gape	0.198	0.723	0.582
Velocity of gape opening	−0.952	−0.125	−0.073
Velocity of gape closing	−0.648	−0.131	0.531
Time to start hyoid depression	0.814	−0.424	0.028
Duration of hyoid depression	0.798	0.339	0.175
Maximum hyoid depression	−0.780	0.421	0.240
Velocity of hyoid depression	−0.898	−0.168	0.071
Maximum head elevation	0.356	−0.538	0.671
Duration of head elevation	0.889	−0.278	0.25
Velocity of head elevation	−0.766	−0.212	0.350
Total variance explained (%)	57	15.5	11.7

### Behavioral variation

To account for behavioral variation, the CV were calculated for the 12 variables for each individual in the four feeding modes. The mean coefficient of variation after correcting for individual variance was 0.18 ± 0.04 (mean ± se) for suction feeding in the aquatic stage, 0.28 ± 0.04 for jaw prehension, 0.27 ± 0.03 for lingual prehension, and 0.26 ± 0.05 for suction feeding in the terrestrial stage (the latter without correction of individual variation because only one individual provided data). The fractional factorial designed ANOVA showed a significant difference between modes (Wilks’ lambda *F* = 239; *P* = 0.003) but not between individuals (Wilks’ lambda *F* = 1.604; *P* = 0.126). The post hoc test, after correcting for individual variation, further revealed that the significant difference between modes was based on significant differences between suction feeding in the aquatic stage and jaw prehension (*P* = 0.002). The CV’s of the other modes showed no significant differences amongst each other.

### Coordination of movements

Regarding timings of gape and hyoid movements, we found significant correlations between the time to peak gape (i.e., duration of gape opening) and the time to peak magnitude of hyoid depression (i.e., time from start gape opening to peak hyoid depression) in suction feeding in the aquatic stage (*r*_25_ = 0.78; *P* < 0.001), jaw prehension (*r*_25_ = 0.93; *P* < 0.001), and tongue prehension (*r*_25_ = 0.94; *P* < 0.001), but not in suction feeding in the terrestrial stage (*r*_5_ = 0.81; *P* = 0.097). Due to the more complex movement patterns in tongue prehension, we further tested for three more time locks between hyoid and gape movements and accordingly found correlations between the time to the first gape peak and the time to the first hyoid peak (*r*_25_ = 0.47; *P* = 0.025), between time to local minimum gape and time to local minimum hyoid (*r*_25_ = 0.69; *P* < 0.001), and between duration of total gape cycle and time to second peak hyoid (*r*_25_ = 0.95; *P* < 0.001). The variable time to local minimum gape further correlated with the time to maximum tongue protraction (*r*_25_ = 0.86; *P* < 0.001) in the tongue prehension mode.

**Fig. 5:**
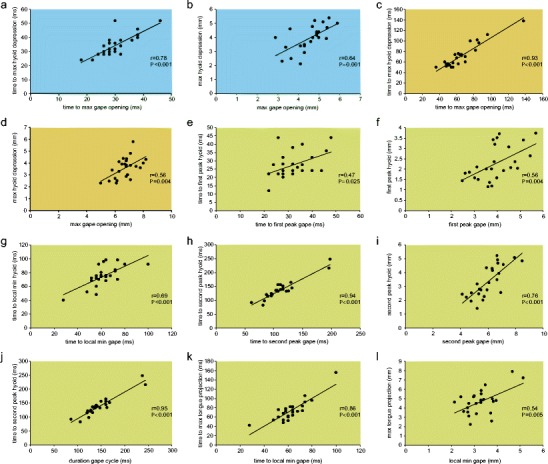
Significant correlation plots of kinematic variables. The feeding modes are color-coded: *blue* (**a**, **b**) suction feeding in the aquatic stage, *light brown* (**c**, **d**), jaw prehension in the aquatic stage, and *green* (**e**–**l**) tongue prehension. Note that the *second peak hyoid* (in **h**–**j**) and *second peak gape* (in **h** and **i**) correspond to maximum hyoid depression and maximum gape opening, respectively

Similar to timing, we further tested for correlations between peak magnitudes of the above listed kinematic variables of gape and hyoid excursions and found significant correlations between magnitudes of maximum gape opening and maximum hyoid depression in suction feeding in the aquatic stage (*r*_25_ = 0.64; *P* = 0.001), jaw prehension (*r*_25_ = 0.56; *P* = 0.004), and tongue prehension (*r*_25_ = 0.76; *P* < 0.001), but not in suction feeding in the terrestrial stage (*r*_5_ = 0.81; *P* = 0.1). In tongue prehension, we further found significant magnitude correlations between the first gape peak and the first hyoid peak (*r*_25_ = 0.56; *P* = 0.004) and between local minimum gape and maximum tongue protraction (*r*_25_ = 0.54; *P* = 0.005), but there was no correlation between local minimum gape and local minimum hyoid (*r*_25_ = 0.17; *P* = 0.42). All significant correlation plots are shown in Fig. 5. In sum, movements of gape and hyoid (plus tongue and gape movements in tongue prehension) might be regarded as “well-coordinated” in suction feeding in the aquatic stage, jaw prehension, and tongue prehension as movements correlate both chronologically and in magnitudes (though in the latter mode, one variable, local minimum gape and local minimum hyoid, did not correlate in magnitude). By contrast, movements of gape and hyoid in suction feeding in the terrestrial stage were not coordinated.

## Discussion

### Flexibility of behavior

Our data showed that *L. vulgaris* is characterized by seasonal flexibility in prey-capture kinematics as previously observed in the closely related Alpine newt *I. alpestris* (Heiss et al. 2013a). The smooth newt responded in a similar way as the Alpine newt to the four feeding circumstances (Fig. 2). Both species used suction feeding by fast mouth opening followed by hyobranchial depression for both underwater strikes in the aquatic and terrestrial stages, as shown here by the analyses of variance and the broad overlap in their kinematic space (Table 2; Fig. 4). When capturing prey on land in the aquatic stage, however, both *I. alpestris* (Heiss et al. 2013a) and *L. vulgaris* (current study) used jaw prehension with a movement pattern roughly similar to suction feeding but nevertheless separated from it in kinematic space with only a small overlapping area (Fig. 4). Interestingly, the main differences between the suction feeding movements in both phases and the jaw prehension mode are related to the fact that jaw opening and hyoid depression were significantly slower during jaw prehension (see Table 2). However, under the same muscle activation conditions, movements like these acting in the direction of gravity should be faster on land because of gravity and the highly reduced fluid dynamic resistance due to the lower density and viscosity of air (Stayton 2011; Van Wassenbergh 2013). This indicates that decreased speed of terrestrial jaw prehension is not a passive result of the different physical properties of water and air, but probably involves different muscle activation patterns compared to suction feeding. When newts had changed to their terrestrial stage, they further modulated their behavior and used tongue prehension to capture prey. During tongue prehension, prey were no longer grasped by the jaws, but were captured by the quickly protruded tongue and brought into the mouth. Accordingly, the latter movement pattern clearly differed from jaw prehension, as shown by the analyses of variance (Table 2) and the small overlapping area in kinematic space (Fig. 4). Altogether, as these kinematic differences between the four feeding modes are now observed for each of the two species studied to date, this suggests that this type of seasonal behavioral flexibility (also referred to as behavioral plasticity (Heiss et al. 2013a)) might be widespread among newts with a multiphasic lifestyle. Newts represent a monophyletic clade (Zhang et al. 2008), but members that do not undergo such dramatic seasonal changes might show a different behavioral repertoire and probably less flexibility.

The ability of the smooth newt to actively change its prey-capture mechanism when changing stage allows a fine-tuning of the prey-capture system to the corresponding habitat in order to maintain performance in both environments. This behavioral plasticity offers great opportunities to exploit food sources from two very different environments where prey availability also varies significantly with seasons (Nöllert and Nöllert 1992; Griffiths 1997). We showed that the smooth newt can also successfully capture prey on land in its aquatic phase by quick changes in its strike movements. Suction feeding in the terrestrial stage, however, seemed to be more challenging and this behavior could only be documented in one individual out of 20 that were available for this study. The reason for reduced tendency to display this behavior is probably not related to functional-morphology of the feeding system, but may be caused by the smooth newt generally avoiding water when in its terrestrial stage. In fact, in our experimental setup all (except one) of our animals immediately left the water when immersed in their terrestrial stage and the food offered under water was ignored. However, in very few cases, other individuals could be observed to occasionally conduct short voluntary dives and to forage under water during their terrestrial stage in their home aquarium. Unfortunately, their aquatic prey capture behavior could not be recorded in their large tank due to the rarity of this behavior and related obvious technical challenges. Anyway, it is very probable that aquatic foraging in the terrestrial stage also occurs occasionally in the wild. In contrast to the smooth newt, the closely related Alpine newt regularly seeks its home waters in the terrestrial stage (Thiesmeier and Schulte 2010; Kopecký et al. 2010) and readily feeds there (Heiss et al. 2013a). Accordingly, it seems that the aquatic-terrestrial transition in the smooth newt incorporates a more fundamental functional morphological change of the whole organism compared to the Alpine newt and might reflect the dominance of the terrestrial stage in the lifestyle of the smooth newt. This becomes more interesting if we consider that both Alpine newt and smooth newt very often live sympatrically in ponds during their reproductive period where they evidently rely on the same food sources (Nöllert and Nöllert 1992; Griffiths 1997). The considerably shorter aquatic stage and the predominant terrestrial lifestyle of the much smaller smooth newt compared to the Alpine newt might reflect avoidance of competition by a behavioral shift toward a slightly different ecological niche (e.g., Gause 1932).

**Fig. 6:**
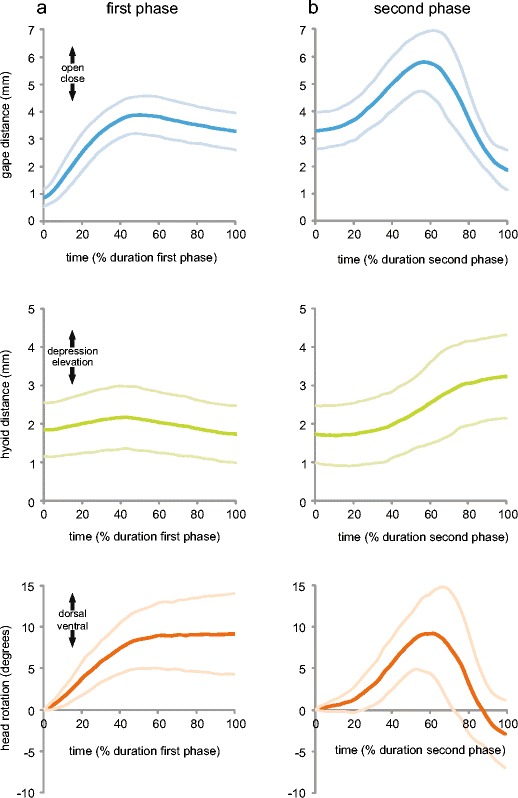
First (**a**) and second (**b**) phase of the tongue prehension mode shown in Fig. 4a. The time axes are normalized to percentages of corresponding phase duration. Both phases can, therefore, be directly compared to the kinematic profiles shown in Fig. 4. Note the striking similarities of movement patterns of the second phase (**b**) and the aquatic feeding patterns shown in Fig. 4a, b, c

Despite obvious morphological and physiological changes during the seasonally induced habitat-shifts in newts, gross-morphological changes of the myoskeletal system of the prey capture apparatus are to our current knowledge unlikely (Heiss et al. unpublished data). Accordingly, the same myoskeletal system has to fulfill very different sets of movements to perform suction feeding and tongue prehension. Based on other studies, many muscles involved in suction feeding are also responsible for the movements used for tongue prehension. However, they differ in activation patterns (Lauder and Shaffer 1988; Deban et al. 2001; Deban 2003), and accordingly, in their neuromotor control. Consequently, the neuromotor control of feeding movements has to change its pattern between aquatic and terrestrial strikes. This alteration in motor control, however, might not rely on a fundamental change, but rather on small modifications and a recombination of existing patterns. In fact, it seems that *L. vulgaris* relies on the same type of stepwise modulation of its ancestral, aquatic prey-capture behavior when changing to the terrestrial mode, as described for *I. alpestris* (Heiss et al. 2013a). In that study, it was argued that tongue-retraction kinematics is probably based on the same motor pattern as suction feeding and that the component of tongue protrusion was added. Similar to the Alpine newt, tongue prehension and suction feeding in *L. vulgaris* are most distinct at first sight and show no overlapping area in kinematic space. However at closer look, the kinematic pattern of tongue prehension can be subdivided into two distinct phases. The first phase comprises movements from the start of mouth opening until the instant of the local gape minimum and the second phase comprises movements from local minimum until the mouth is closed (Fig. 6). The second phase with tongue retraction shares some obvious similarities with the movement pattern typical for suction feeding, and accordingly, it might be assumed that the second phase of the tongue based capture mode is evolutionary derived from suction feeding. Perhaps even the motion of the jaws and hyolingual system during tongue protrusion could be evolutionary derived from the preparatory action observed in many fish and salamanders during which they decrease the mouth cavity volume by protracting their hyobranchial apparatus prior to generating suction (Reilly and Lauder 1989; Lauder and Reilly 1994; Konow and Sanford 2008; Konow et al. 2008). Tongue prehension is assumed to be most effective for terrestrial prey capture in salamanders because the whole body does not have to be accelerated towards the prey as it is the case in jaw prehension. In fact, a rapidly protruded tongue quickly bridges the gap between predator and prey and is used by virtually all terrestrial salamanders (Özeti and Wake 1969; Larsen and Guthrie 1975; Dockx and De Vree 1986; Reilly and Lauder 1989; Findeis and Bemis 1990; Miller and Larsen 1990; Reilly 1996; Deban 1997, 2003; Wake and Deban 2000).

### Stereotypy of behavior

We hypothesized that prey capture movements in *L. vulgaris* might be more stereotyped when feeding in the prevalent feeding modes compared to the reciprocal. This hypothesis was partly supported as suction feeding in the aquatic stage was indeed significantly more stereotyped than jaw prehension. The increase of behavioral variability when newts switched from suction feeding in the aquatic stage to the terrestrial jaw prehension mode is probably based on the fact that animals are fine-tuning their behavior to the abruptly changed mechanical circumstances. Similarly, it might be argued that a comparable scenario with highly variable prey-capture behavior might occur during the very first steps of an aquatic to terrestrial transition in newts with multiphasic lifestyle. During such a phase of highly variable prey-capture kinematics, newts may be modifying a preprogrammed (feed-forward controlled) motor pattern of suction feeding by sensory feedback, in which information of the external environment is transmitted to the central nervous system (Deban et al. 2001). This could result in a more variable behavior as an adaptation to a new environment. However, no evidence was found that this decreased stereotypy also applies to animals in their terrestrial stage that are persuaded to feed underwater. The lack of a significant difference of behavioral variation between the three other modes might indicate that alteration of behavioral variability plays a minor role in other than aquatic-terrestrial transitions.

### Coordination of movements

A coordinated interplay of jaw and hyobranchial movements is advantageous for successful prey capture (Ferry-Graham and Lauder 2001; Wainwright et al. 2008). Accordingly, we hypothesized that jaw and hyoid movements would be well-coordinated despite the lack of mechanical coupling of these systems in newts. In fact, the myoskeletal system of jaws and hyobranchial apparatus are the main elements of both aquatic and terrestrial prey capture in salamanders (Findeis and Bemis 1990; Deban and Wake 2000; Wake and Deban 2000; Deban 2003) but perform a different set of movements between water and air. The movement pattern of fast jaw opening resulting from dorsal skull rotation and lower jaw depression, followed by pharyngeal volume expansion based on hyobranchial depression, is the typical pattern for aquatic strikes, i.e., suction feeding. Such an anterior-posterior expansion wave is hydrodynamically advantageous for suction feeding as shown in a variety of aquatic predators (Muller and Osse 1984; van Leeuwen and Muller 1984; Lauder 1985; Lauder and Shaffer 1986; Van Damme and Aerts 1997; Ferry-Graham and Lauder 2001; Lemell et al. 2002; Kane and Marshall 2009; Herrel et al. 2012) and the movements of jaws and hyoid are in fact well-correlated in *L. vulgaris* when suction feeding in the aquatic stage. In contrast, when suction feeding in the terrestrial stage, movements of jaws and hyoid are not correlated which might reflect the difficulties of animals in the terrestrial stage to quickly switch to suction feeding in a well-coordinated way. Analogously, coordination between jaw and hyoid movements might be relatively low during the very first steps of a terrestrial to aquatic transition but increase with time and “practice.”

In contrast, jaw prehension shows well-correlated movements of the jaws and the hyobranchial system. Accordingly, the jaw prehension mode should not be viewed as uncoordinated and random capture trials but as a distinct and well-coordinated prey capture mode that relies on an actively modified suction feeding pattern. The suction feeding pattern is further modified for tongue prehension after animals have changed to their terrestrial stage. The movement pattern becomes more complex with essentially two magnitude peaks both regarding gape and hyoid movements with a local minimum in between (Fig. 3d). Interestingly, not only jaw and hyoid movements are tightly coordinated despite the relatively complex movement pattern, but also tongue movements are well-coordinated with jaw movements.

### Evolutionary implications

Tetrapods evolved from sarcopterygian fishes (e.g., Carroll 2009) and sarcopterygians use suction feeding to capture prey (Lauder 1985; Bemis and Lauder 1986). Consequently, suction feeding is generally assumed to be the ancestral prey-capture mode in tetrapods (Lauder 1985) from which primitive terrestrial capture modes, namely grasping prey by the jaws and later on more elaborate, tongue-based prey-capture modes, evolved secondarily. The mechanisms behind such significant behavioral transitions are still poorly understood, mostly due to the lack of proper extant model organisms. Our findings corroborate the hypothesis that a new behavior required for an aquatic-terrestrial transition might not rely on a dramatic reorganization of the ancestral, i.e., aquatic, motor pattern but rather on slight modulation and recombination of an existing pattern. This hypothesis was put forward in studies of the locomotor system showing that even small changes of the neuromotor control of movements can lead to different movement patterns, which can be tuned to respond the new demands of a changed environment. For example, Ijspeert et al. (2007) and Knüsel et al. (2013) showed that central pattern generators (i.e., neural networks setting the basic patterns of repeated motor activities) can successfully generate coordination patterns for swimming and terrestrial walking in salamanders with only minor modifications. Analogously, we hypothesize that only small changes in the neuromotor program that controls feeding movements are needed to change from the aquatic feeding pattern, i.e., suction feeding, to the terrestrial patterns, i.e., jaw prehension, and further on to tongue prehension.

Similar to the stepwise modification of the suction feeding pattern as response to an aquatic-terrestrial transition in newts, it is not unlikely that a small change of the neuromotor control in early tetrapods has led to a slightly changed feeding behavior to allow prey capture on land and the invasion of terrestrial niches to exploit new food sources there. Such a scenario might be corroborated by other extant analogs: amphibious fishes with the ability to feed on land actively adjust their prey capture behavior and in fact use slightly modified aquatic prey capture movements to feed on land (Sponder and Lauder 1981; Van Wassenbergh et al. 2006; Van Wassenbergh 2013). Similarly, intraoral food processing (e.g., chewing) which relies on a rhythmic pattern of coordinated jaw and hyoid movements might have been altered during the fish-tetrapod transition to account for the new mechanical demands by only a small change of the muscle activity pattern (Konow et al. 2011).

## Conclusion

Newts offer a unique opportunity to analyze the functional constraints behind aquatic-terrestrial transitions. Our results indicate that a high degree of seasonal kinematic flexibility of the prey-capture system is not uncommon in newts. This flexibility involves a seasonal loss and gain of the capacity to capture prey through prehension by the tongue, which from a neuromotoric point of view, probably require only relatively subtle adjustments to the ancestral suction-feeding motor program. The relatively short duration of the aquatic stage of our model species *L. vulgaris* does not interfere with this seasonal flexibility, but may explain why underwater feeding when the animals are in the terrestrial stage was difficult to elicit. The precise changes in the neuromotor control between stages and how these changes arise in intermediate steps (i.e., while shifting habitat) currently remains unresolved. As this information would further our understanding of the processes involved in environmental transitions, this may be the focus of future studies.
